# Case Report: A Functional Approach to Deal With Sexual Harassment Within Families

**DOI:** 10.3389/fpsyg.2022.537599

**Published:** 2022-05-31

**Authors:** Li Meiling, Alishba Hania, Muhammad Waqas

**Affiliations:** ^1^School of Economics and Statistics, Guangzhou University, Guangzhou, China; ^2^Institute of Southern Punjab, Multan, Pakistan; ^3^Department of Business Administration, Institute of Southern Punjab, Multan, Pakistan

**Keywords:** sexual harassment, school counselling, functional analysis, emotional assessment, crisis management

## Abstract

Abuse is followed by multiple short- and long-term psychological consequences. Researchers suggest a significant need to design a culturally relevant and competent treatment plan specifically for the Asian context. This research presents an account of therapeutic strategies that were employed to manage the complaints of sexual harassment and associated psychological challenges. This case report dealt with a 16-year-old girl who was self-referred to the psychologist with complaints of getting sexually harassed by her brother-in-law, difficulty in managing academic challenges, communication gap with parents, and difficulty in handling her anger for the past 7 years. The assessment modalities used with the client were Behavioral Observation, Clinical Interview, Baseline Charts, School Children Problem Scale to rule out emotional–behavioral problems, the Adult–Child Interaction Test, and Downward Arrow Technique for cognitive assessment. The management plan was based on trauma-focused cognitive behavioral therapy (TF-CBT) and constituted of goals, such as having self-protection awareness from harassment, reducing the fear and unexplained emotions of grief, increasing self-esteem, designing a proper schedule for studies, and developing good communication ability. Post-assessment showed that anger reduced to 40%, communication gap with parents reduced to 50%, fear of harassment reduced to 40%, and self-image improved up to 40%. Sessions were based on 9 in-person meetings.

## Introduction

Sexual harassment is a challenge for both developed and developing countries worldwide (Drobac and Russell, 2020). Although the exact number of victims is still unknown because of a lack of accurate reporting (Stoltenborgh et al., 2011), sexual harassment is comparatively a gender-specific issue as the majority of cases are women (Essar et al., 2021). Studies have debated that sexual abuse in early life is significantly associated with the psychological and economic burden on the individual, the family, and the community. Previously, sexual abuse and associated challenges were primarily managed by cognitive behavioral therapy (CBT), and medications had a secondary part (Hetzel-Riggin et al., 2007). However, the effectiveness of these interventions has only been tested in individualistic cultures, namely, the West. Pakistan is a Muslim country and, culturally, it is expected from the young generation to control their sexual impulses and actions. Moreover, the conversation on sexual issues is marked with shame and denial.

Pakistani culture is characterized as a collectivistic one that prioritizes establishing harmony in the whole family over the needs of one person even if the matter is as sensitive as sexual harassment of minors (Tang, 2002). If the report of abuse apprehends a breakup in the family structure or brings a bad image to the family, the disclosure is discouraged. The help of mental health professionals is only sought when victims show extremely severe emotional/behavioral symptoms, significant academic decline, self-harm or suicidal tendencies, or symptoms of conversion disorder, such as feeling supernatural possession accompanied with loss of consciousness. At times, families of victims are unaware of the abuse, and perpetrators are often within the family which further complicates the management.

The ultimate goal of a therapist is to help the victim move forward with effective coping mechanisms of survival from the past experiences, and it is done by drawing the client’s attention to the strengths and innate capacity to adapt which ultimately induces hope and optimism (Zadafshar et al., 2021). Consequently, self-esteem is improved, and academic and social activities are reestablished and reassimilated. The sole purpose is to empower the survivor to consider future possibilities of living a better life. Simultaneously, the therapist is required to involve the families in aiding to adjust the client to traumatic experiences. However, in this case report, the family was not approached due to the cultural restrictions and the client’s present ability to share the trauma. It is interesting to know that researchers have found scenarios in which the client was able to move forward from the trauma, without any professional help taken (Hetzel-Riggin et al., 2007). The resilience of a person plays a significant role in determining the prognosis of a client. In this case report, we opted for an individualistic approach with a cognitive-behavioral approach (McDonagh et al., 2005) to manage the reported presenting complaints of the client, and positive adaptation to the trauma was followed. Although trauma-focused CBT (TF-CBT) is a family focused approach, family dynamics in a collectivistic Muslim society, such as Pakistan, are different, and elements, such as shame and guilt, have to be addressed in the families which takes comparatively longer duration and energy of the survivor. A positive therapeutic alliance is proposed to be as operative as an emphasis on narration in transitioning the survivor to adaptive functioning.

## Case Report

Problems reported by the client were getting sexually harassed by a brother-in-law, difficulty in maintaining academic challenges, having a communication gap with parents, having fear of getting harassed again, and having difficulty in handling own anger from the past 7 years. When asked about her daily routines and likes and dislikes, it was stated that “I love watching cartoons, TV dramas of family TV channels and playing video games online.” She had no more than two friends at school but even with them, she shares limited information about her feelings. The client was seemingly good at understanding the educational concepts and reported *I learn things quickly in class if I am not stressed but when thoughts of past harassment are triggered, I start forgetting things in class and at home.* She had a sharp memory and sense of humor that could be highlighted as some of her strengths because she kept sharing new jokes that she read in the daily newspaper or heard from someone in class. In the future, the client aims to do a Nazra course (learning Quran from a mosque) but when inquired about the inspiration, she said, *There are no boys in this field, and I will be safe*. The client belonged to a middle-class family based on 3 members who were her parents and herself. The mother of the client is uneducated, whereas her father had passed his graduation when he got married. The client reported, *My father takes all the decisions at home, and everyone is scared to disobey whatever he says and sometimes I cry while hiding but eventually do what pleases him*. The client’s family is highly attached to Islamic institutions and preaches religious teachings. Her father always had people come to their home and sit in the drawing room, and most of those people were men; therefore, the client mostly stayed in her room instead of roaming around in the house. The client’s father is a 61-year-old man who was a retired government school teacher and her mother was a housewife. Sister-in-law and brother-in-law were married by the father’s choice. The client reported, *My father never listens to me and gives me a shut-up call because my brother-in-law has told him that I am a disobedient girl and words of men are trusted more than girls in my home*. The client’s father is the only earning hand in the house and pays for all the members of the house, while the brother-in-law does not have a job and there is no other man in the house. The client reported that her early education was given by her father that was based on Quran and Sunnah. At the age of 4 years, she was admitted to the school near her house. The client is reported to be a good student and fast learner until she passed 3 classes. Then, her performance started to become average.

The detailed history revealed, factors that predisposed the client’s trauma, were emotional and communication distance from the family that had worsened her problem. The client has come with problems of feeling fearful regarding her harassment, low academic performance, and grief on losing contact with a teacher who gave her most attention in the class. The precipitating factor, in this case, was the parent’s lack of trust in her and the conspiracy about her character created by her brother-in-law who was himself involved in harassing her. A maintained factor in this case that strengthened the problem was the continuously challenged academic performance of the client.

## Assessment

### Reinforcer Identification

Reinforcer identification was followed to develop the rapport and to use these reinforcers in strengthening her motivation toward managing her problems (Table 1; Mintz et al., 2007). This phase was achieved during the initial rapport building. These were inquired by asking likes and dislikes of the client and what she did in her leisure time.

**TABLE 1:** Priority-wise reinforcers of the client along with categories.

Categories	Reinforcers
Activity reinforcers	Watching favorite drama on TV Playing new online games Writing Diary
Social reinforcers	Spending spare time with mother and learning to cook Getting praised by teachers on scoring good marks

### Baseline Charts

ABC charts of anxiousness, anger, and guilt were maintained (Table 2). The purpose of these charts was to assess the triggers and thoughts behind them (Miltenberger, 2015). The client was asked to identify events and triggers and then to report the thoughts and feelings, followed by consequences in terms of emotions and behaviors. Through these charts, the client’s thoughts were assessed in different situational triggers, and some of the cognitive errors that were noticed were personalization, filtering, overgeneralizing, and jumping to conclusions. ABC charts were maintained to understand the triggers of the behavior and to rule out the thoughts and feelings. This was done with the client to assess the triggers of anger and also to understand the reported guilt (Miltenberger, 2015).

**TABLE 2:** Functional behavior analysis of anger and guilt.

Problem	Average frequency	Average intensity	Average duration
**Anger**			
Session 1	8 times/day	5/day	30 min/day
Session 9	3 times/day	4/day	5 min/day
**Guilt**			
Session 1	7 times/day	8/day	10 min/day
Session 9	0 times/day	0/day	0 min/day

The baseline charts indicated that the client felt angry whenever she sees her brother-in-law or see any male who was not paying attention to her and even her father. This anger then converted into guilt feelings and made her feel sad about herself. In addition, she rated her anger on 9 and guilt on 7. She further reported that it usually lasts for one or half hours on average. According to the client, her anger resolves when she talks to her mother, listens to songs, plays video games, or gets praise from her mother.

### Subjective Ratings

Initially, to assess and then monitor the progress of the management, subjective ratings were taken from the client about the target problem (Hayhurst et al., 2015). The client was also asked to rate her anger, feeling of guilt, fear of harassment, communication challenges with parents, the value you assign to yourself as a person (self-worth), and academic challenges. The marks were distributed in the form of ratings from 1 to 10, where high scores determined high difficulty. Table 3 shows the ratings.

**TABLE 3:** Subjective ratings of anger and guilt, fear, and emotional distance from mother.

Client problems	Sessions	Average subjective ratings
Anger	Session 1	9
	Session 9	6
Guilt	Session 1	7
	Session 9	4
Fear for harassment	Session 1	10
	Session 9	4
Communication challenges with parents	Session 1	6
	Session 9	2
Self-worth	Session 1	2
	Session 9	6
Academic challenges	Session 1	3
	Session 9	7

The subjective ratings depicted that the client had high ratings in the initial sessions of the presented complaints. With the start of management techniques, the ratings gradually decreased.

### Emotional–Behavioral Assessment: School Children Problem Scale

The School Children Problem Scale (SCPS) (Saleem and Mahmood, 2012) was administered due to the reported complaints of anger and anxiousness regarding academic challenges. SCPS explained the dimensions of emotional and behavioral problems. The instructions were given to the client, and she rated the statements according to a 4-point Likert scale before and after the intervention. The interpretation of the quantitative result is shown in Table 4.

**TABLE 4:** Scores, mean, standard deviation, and categories on School Children Problem Scale.

Factors	Obtained scores	Mean	Standard deviation	Category	Post-assessment scores	Category
Anxiousness	18	13.48	7.28	v.severe	10	Mild
Academic problems	12	7.11	4.42	v.severe	6	Moderate
Aggression	10	7.05	4.35	v.severe	4	Mild
Withdrawal	16	8.51	3.94	v.severe	9	Moderate
Rejection	7	3.30	3.25	v.severe	3	Mild
Somatic complaints	6	4.45	19.71	severe	3	Mild

Significantly high scores were observed in all the domains that indicated severe emotional and behavioral problems, keeping in view the negative experiences of the client. The client had withdrawn from her daily activities and interests probably because of the trauma she had faced combined with the lack of communication with her parents. Perceived rejection is severely reported. We observed from the scores that the client was experiencing severe emotional and behavioral problems. Moreover, with the unavailability of a proper outlet for emotional turmoil due to trauma, she had high scores on passive aggression.

### Downward Arrow

The overall systematic assessment revealed problematic areas. For further probing of deep-down negative thoughts and beliefs, it was necessary to rule out the core beliefs of the client therefore downward arrow (Leahy, 2008) was done. Downward arrow revealed that the core belief of the client was to consider herself worthless and only consider herself powerful when she had others’ acceptance for her behaviors and feelings.

### Adult–Child Interaction Test

It is a projective test developed by Alexander (1952) consisting of eight cards showing pictures of children and adults. Two main divisions for analysis are apperception and reasoning and motivation and emotion. It was planned to primarily get information about the perceptive experience of the client about adults and how she perceives adults’ perceptions of her. Moreover, to assess the child’s interaction with adults, she seemed to be defensive in disclosing her reaction to family.

### Summary of Assessment

The assessment results revealed that the client is experiencing significant academic and emotional challenges along with a distorted image of self and authority figures. The client has negative core beliefs about herself which are affecting her morale to achieve her goals or move forward and cope with the trauma of sexual harassment.

## Intervention Plan

### Rapport Building

It was the initial step for management. It involved the process of establishing a trustworthy relationship with the client by engaging the client in her desired activities and assuring confidentiality (Hershkowitz, 2011). Initially, the client was not able to verbalize her challenges and history of sexual trauma. The clinician adopted an empathetic attitude of acceptance and relayed her sentiments stating, *I understand why you are here, and we can talk about anything you want to share. I am here to help you and I want you to trust me*. The client was allowed to speak without interruptions and was not stopped from crying and expressing her sadness and was shown empathy. Over the next few meetings, the client started sharing information about the sexual harassment she had faced. Summarizing and paraphrasing were used in maintaining the rapport, and focused listening was adopted throughout the sessions.

### Psychoeducation

It was the procedure in which the client was educated about her condition, its causal factors, and management plans that could be utilized, including the protective factors. Protective factors and case conceptualizations helped in relapse prevention. It was done to explain to the client about the course of counseling sessions along with the therapist’s role and the effectiveness of client’s cooperation. In addition, she was provided the rationale of the management plan devised for her problems. The clinician tried to prepare the client on responding to certain symptoms along the way, “What should we do if this happens?”

### Self-Protection Awareness From Harassment

Among various people who get sexually harassed, one of the most common things that arises is self-blame (Ahmed et al., 2019; Adhikari and Husain, 2021). Therefore, the client was assured about her victimized state and what happened was done and her effort to come forward and show a willingness to cope was appraised. The client was told about the statistical number of girls who were being harassed sexually in our country to let her know there are other people too and she is not the odd one out. Moreover, she was appraised that not all those girls come forward to talk about the harassment they have witnessed. She was told that sexual assault and abuse do affect you, but it does not define who you are. Healing and recovery are possible, and we will be talking about coping strategies and resources in future sessions to help you in this process.

#### Deep Breathing and Relaxation Techniques

For future reference, the client was helped through role-plays regarding keeping her mind in control for taking quick decisions instead of getting horrified and losing control of the situation. The client practiced deep breathing and positive motivational statements in mind to prepare herself for the situation when her brother-in-law (the perpetrator) approaches her because she used to become very anxious at the time. The client was educated about the physiological impacts of threats that act in a cycle and stop the mind from working straight; therefore, she practiced taking over physiological breakdowns in threatful situations. The client was taught to use the skills of relaxation, such as deep breathing, grounding, and mindfulness, when she feels worried about something bad happening in near future. She was taught to focus on a second at a time to help her ground at the moment.

#### Crisis Management

For management and getting the client prepared for the crisis, the following steps were taken as suggested by Cohen (1988). First of all, the client was given a scenario of another girl who is in her house being harassed just like her; what will be the safety measures she could take in that situation and some factors were identified, such as shouting for neighbors if nobody is at home, separating things that can hurt or push the perpetrator away, locking the door of the room when alone at home, running toward washroom and closing the door behind so that she cannot be followed, and recording the voice of brother-in-law while he tries to harass again. Second, statements were rehearsed for the realization that the abuser is the one at fault. We designed a priority list from least to most, based on people who could be called, and their numbers were saved on speed dials. She was also made to learn phone numbers of close-by neighbors to call in an urgent situation.

Another powerful part of the intervention was preparing this client for trauma narrative as suggested by TF-CBT approach. Because of the prolonged trauma, the first draft comprised 22 handwritten pages. Therefore, she was instructed to write the “first, worst, and the last.” The narrative was written in the presence of the psychologist but in a side of the counseling room where she remained undisturbed and she was occasionally asked, *Do you need me*. The session targeted that narrative building exercise would contain 10–15 min, in the end, to discuss how the client felt writing this narration while occasionally challenging her cognitions. The challenging cognitive distortions were also identified and she was taught “thought stopping and displacement techniques” to counter them. The last section was targeted on *How do you see yourself in the future? Is it different now that you have been through this experience?*

### Reduce the Feeling of Grief and Unexplained Emotions

As the client had been a victim for a long time along with a suppressed child in the family who had been taught to not express her emotions in front of elders. The rationale behind this was to bring her back to her optimum coping potential (Deblinger et al., 1999). At first, the client was educated about the consequences of not expressing the jumbled emotions and their psychological and physiological impacts. The client was provided empathy and active listening to let her express her sadness and grief regarding harassment. She was told that our unexpressed emotions also turn into anger, fear, and behaviors of damaging things as well as avoid things of our interest, such as positivity in relationships, and focus on studies. In addition, she was told about the connection of feelings with thoughts which brings bodily and behavioral changes. For bodily changes, deep breathing was taught, and thoughts were dealt later through cognitive restructuring.

#### Cognitive Restructuring

It was done using several techniques, as described below. The client’s belief identified through the downward arrow technique was dealt with evidence for and against the technique. Later, the client assigned scores for and against beliefs and concluded that her thoughts were not based on reality and were distorted due to negative experiences. Alternative thoughts and ideas were brainstormed, and the client concluded that she could achieve great things but it was only limited because of her practice of discounting the positives from her life. The client felt confident to deal with the possible threat of getting harassed again and showed compliance in rehearsing the possible scenarios.

### Communication Challenges

To deal with passive aggression and the inability to communicate with parents, the client was taught the following techniques.

#### Assertive Training

The client practiced positive and negative inquiries in a decent way that utilized respectful words and sentences that had a limited sense of disrespect in them (Rigby, 2007).

*Systematic desensitization* was used to deal with the behavioral avoidance of the client. In this technique, hierarchies of interaction with parents were made by probing the least threatening encounter with them (Curran and Gilbert, 1975). Moreover, deep breathing was included to utilize the principle of reciprocal inhibition, and anxiousness was replaced by relaxation. The least threatening situation for the client was when her mother is usually free after mealtime in the afternoon and plays games on her mobile. The client was asked to start a friendly conversation with her from that time and was taught to include mother time in her daily routine chart. This was done to strengthen her bond with her mother and to have her as a saving mediator in threatening situations. The client reported to start building a bond with her mother as her father was mostly outside. She had started sharing her daily routine with her and used positive statements while interacting with her.

### Self-Esteem Building

The assessment revealed that the client perceives herself as a rejected entity, and because of that, she had low self-esteem that led her to the symptoms of withdrawal.

*Psychoeducation with the CBT model* was used in explaining how our thoughts are related to our actions, and she was asked to explore alternative thoughts in similar scenarios.

#### Positive-Coping Statements

The client was asked to list down her strengths to recall them every time she had negative automatic thoughts about the events, she is in. Cards were prepared with positive statements, and she kept them nearby (Goldstein and Brooks, 2005; Gooding et al., 2016).

### Time Management

It was done to help the client concentrate on her studies and spend her spare time in useful activities instead of recalling her negative experiences (Marais et al., 2020). At first, a self-reinforcement plan was made with the client to enlist what possible reinforcers she will be giving herself after achieving the goals she had made for herself every week regarding studies.

## Outcome

**FIGURE 1:**
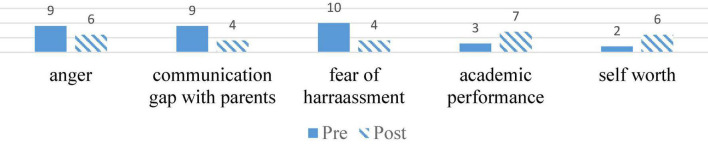
Pre- and Post-assessment by Psychologist.

Figure 1 shows the pre- and post-management ratings for all the presenting complaints were taken based on the subjective evaluation of the client.

## Discussion

In the case discussed earlier, some of the neglected factors that are responsible for determining a positive prognosis of any client with an experience of sexual harassment are discussed. The whole process of therapy consisted of 9 in-person sessions with the client in the school setting. In this process, assessment modalities of a single case study provided in-depth information about the predisposing, precipitating, and maintaining factors of the reported emotional and behavioral challenges. Although scarcely reported, estimated cases of abuse are 9% among girls and 3% among boys worldwide (Barth et al., 2013), but the rate is varying depending on the culture, definition of abuse, and harassment in a certain culture and the research methodologies. Abuse and harassment among children and, especially, girls are extremely underreported (Reitsema and Grietens, 2016).

In Pakistan, sexual harassment and sexual abuse are the lowest recorded crimes. However, as the media highlighted the cases of 6-year-old Zainab, 10-year-old Farishta, and 2019’s Kasur case of 280 abused cases, the public has started addressing these issues in a new light, and victims have started coming out with their verbatims showing an annual increase in reports of child abuse (Abbas and Jabeen, 2021). Factors, such as gender discrimination and lack of awareness about the rights of children, have reinforced the ideology of keeping girls restricted within the confines of their homes to protect them from strangers. However, majority of sexual harassment cases worldwide show the perpetrator to be among close family members (Gekoski et al., 2016). Moreover, in Pakistan, recent research showed the most reported perpetrators to be parents followed by friends and teachers (Abbas and Jabeen, 2021).

The intervention plan of this research was aimed to manage the impact of sexual harassment in the form of passive aggression and guilt, and the associated maintaining factors, such as academic challenges and communication gap with significant family members, namely, parents of the client. The management plan was constituted of trauma-based cognitive and behavioral techniques that are known to be highly effective with cases of sexual harassment (Lewey et al., 2018). The results were noted in the form of subjective reports and the assessment measures, and the problems showed a significant improvement. In addition, pre- and post-ratings were compared, and the results validated the effectiveness of our treatment plan. The follow-up in-person sessions were not continued because the family had to relocate to a different city. The psychologist continued the follow-up with the client through phone calls once a month and, after 3 months, the client was referred to a new school psychologist.

## Treatment Implications

This case illustrates an example that a manualized treatment can be used in a school setting. The psychologist was under the supervision of a trained TF-CBT expert and had more than 4 years of practice in managing sexual abuse. In this case report, TF-CBT to a client was not intellectually mature enough as an adult and emotionally challenged due to past traumatic experience of sexual harassment. This demonstrates the wide applicability of this treatment approach and adds to the pieces of evidence with a diverse clientele. Some limitations were faced during the treatment, given the client’s limited cognitive knowledge. The client faced challenges in understanding some cognitive concepts and their utility, such as thought replacement. The psychologist worked hard and explained the concepts a few times until she understood them. Often the client seemed frustrated or confused but the psychologist encouraged her to take breaks, practice deep breathing techniques, and later review the concepts again.

## Future Recommendations

The practitioners who wish to work with victims of sexual harassment must learn a great deal about TF-CBT. There is a significant need to modify the language used to describe such victims in the cultures, namely, Pakistan. All the terms that degrade the victim or blame the victims must be highly discouraged. A psychologist must view the client as a person in need of growth. The step that is deemed critical for success and most important in this study is the phase of rapport building between the client and the psychologist. Key points to follow to build this rapport and make the client open up are to know the client as a person and understand her likes and dislikes.

## Conclusion

The case report intends to propose a holistic approach of dealing with victims of sexual harassment within the family. The case is of great importance as it illustrates a detailed management plan for managing sexual harassment and associated psychosocial challenges in an individualistic culture. The goals of management were to enable the client and deal with traumas of harassment through TF-CBT. Overall, this case study is proposed as an extensive piece of literature providing an effective intervention plan. However, the limitation of this study is limited generalizability.

## Data Availability Statement

The datasets analyzed in this manuscript are not publicly available. Requests to access the datasets should be directed to MW, mwaqas@mail.ustc.edu.cn.

## Ethics Statement

The studies involving human participants were reviewed and approved by the Ethics Committee at the Guangzhou University and Institute of Clinical Psychology, University of Management and Technology, Lahore. Written informed consent to participate in this study was provided by the participants’ legal guardian/next of kin. Written informed consent was obtained from the minor(s)’ legal guardian/next of kin for the publication of any potentially identifiable images or data included in this article.

## Author Contributions

All authors equally contributed to conception and design, acquisition of data, analysis and interpretation of data, drafted the article for important intellectual content, approved final version to be published and agreement to be accountable for all aspects of the work in ensuring that questions related to the accuracy and integrity of any part of the work are appropriately investigated and resolved.

## Conflict of Interest

The authors declare that the research was conducted in the absence of any commercial or financial relationships that could be construed as a potential conflict of interest.

## Publisher’s Note

All claims expressed in this article are solely those of the authors and do not necessarily represent those of their affiliated organizations, or those of the publisher, the editors and the reviewers. Any product that may be evaluated in this article, or claim that may be made by its manufacturer, is not guaranteed or endorsed by the publisher.
